# Targeting CMV Reactivation to Optimize Care for Critically Ill COVID-19 Patients: A Review on the Therapeutic Potential of Antiviral Treatment

**DOI:** 10.3390/v15051165

**Published:** 2023-05-13

**Authors:** Georgios Schinas, Vasiliki Moustaka, Eleni Polyzou, Maria Panagiota Almyroudi, George Dimopoulos, Karolina Akinosoglou

**Affiliations:** 1 Medical School, University of Patras, 26504 Patras, Greece; georg.schinas@gmail.com (G.S.); polyzou.el@gmail.com (E.P.); akin@upatras.gr (K.A.); 2 Medical School, National and Kapodistrian University of Athens, 11527 Athens, Greece; v.moustaka33@gmail.com; 3 Department of Internal Medicine and Infectious Diseases, University General Hospital of Patras, 26504 Patras, Greece; 4 Department of Emergency Medicine, University Hospital ATTIKON, Medical School, National and Kapodistrian University of Athens, 12462 Athens, Greece; mariotaalm@yahoo.gr; 5 3rd Department of Critical Care, EVGENIDIO Hospital, Medical School, National and Kapodistrian University of Athens, 11528 Athens, Greece

**Keywords:** cytomegalovirus infections, COVID-19, antiviral agents, intensive care units, SARS-CoV-2, CMV, critically ill

## Abstract

Cytomegalovirus (CMV) reactivation has been linked to adverse clinical outcomes in critically ill patients, with emerging evidence suggesting a potential connection with severe COVID-19. Mechanisms driving this association may include primary lung injury, amplification of systemic inflammation, and secondary immunosuppression. Diagnostic challenges in detecting and assessing CMV reactivation necessitate a comprehensive approach to improve accuracy and inform treatment decisions. Currently, there is limited evidence on the efficacy and safety of CMV pharmacotherapy in critically ill COVID-19 patients. Although insights from non-COVID-19 critical illness studies suggest a potential role for antiviral treatment or prophylaxis, the risks and benefits must be carefully balanced in this vulnerable patient population. Understanding the pathophysiological role of CMV in the context of COVID-19 and exploring the advantages of antiviral treatment are crucial for optimizing care in critically ill patients. This review provides a comprehensive synthesis of available evidence, emphasizing the need for additional investigation to establish the role of CMV treatment or prophylaxis in the management of severe COVID-19 and to develop a framework for future research on this topic.

## 1. Introduction

Human cytomegalovirus (CMV) is a herpesvirus that affects a significant portion of the global population, with 83% of adults worldwide reportedly having contracted the virus. Transmission occurs through close or sexual contact [1]. Primary CMV infection typically results in mild or no clinical symptoms, but it establishes a life-long latent or persistent infection from which reactivation may occur throughout an individual’s life [2], especially during periods of the host’s immunosuppression [3] or critical illness [4]. Emerging evidence links CMV to cancer, chronic inflammatory diseases [5], and most recently to an increased risk of severe COVID-19, both in the form of seropositivity and reactivation, suggesting a possible interaction between the two conditions [6,7].

The relationship between CMV and COVID-19 has come under scrutiny, as it is unclear whether reactivation is a direct consequence of SARS-CoV-2 infection, COVID-19 treatment strategies such as corticosteroids or other immunomodulatory therapies (e.g., tocilizumab), or if it affects all patient categories (e.g., women, patients on steroids, elderly, immunocompromised) uniformly [8]. This uncertainty implies that CMV reactivation may in fact contribute to immune dysregulation in COVID-19 patients, exacerbating disease severity [9]. Emerging evidence on the decreased short and long-term efficacy of COVID-19 mRNA vaccine in geriatric and health worker populations with latent CMV infection further exemplify a potential association between CMV seropositivity and dysregulated immune response against SARS-CoV-2 [10].

In this context, it is essential to contemplate the use of antiviral treatments for critically ill COVID-19 patients with confirmed reactivation or provide prophylaxis for seropositive individuals with severe COVID-19. While antiviral medications are widely accessible, their clinical application in CMV-complicated COVID-19 remains largely unexplored due to the absence of randomized studies in the current literature. This is not entirely unexpected, considering the limited data available on the efficacy and safety of CMV antiviral therapy in critical illness, with only three randomized clinical trials serving as a basis. Nevertheless, the insights gained from CMV reactivation in non-COVID-19 critical illness can serve as a starting point for discussing the potential role of CMV treatment or prophylaxis in severe COVID-19 cases.

The purpose of this review is to comprehensively summarize the literature on the role of secondary CMV infection in the ICU setting as a marker or contributor to adverse COVID-19 outcomes. By exploring biologically plausible mechanisms of reactivation in COVID-19 critical illness, evaluating diagnostic methods, and synthesizing the results from the studies available, we aim to address the potential utility of prophylactic or therapeutic antivirals in the management of severe COVID-19, and at the same time, establish a framework for future research into the topic.

## 2. Methods

A narrative review was conducted to synthesize and evaluate the existing literature on cytomegalovirus (CMV) pharmacotherapy in critically ill COVID-19 patients. To find relevant articles, we conducted systematic searches in three databases: PubMed, Cochrane Library, and Scopus. We used a combination of keywords and Medical Subject Headings (MeSH) terms related to COVID-19, cytomegalovirus (CMV), antiviral agents, and critical illness in our search strategy.

**Figure 1:**
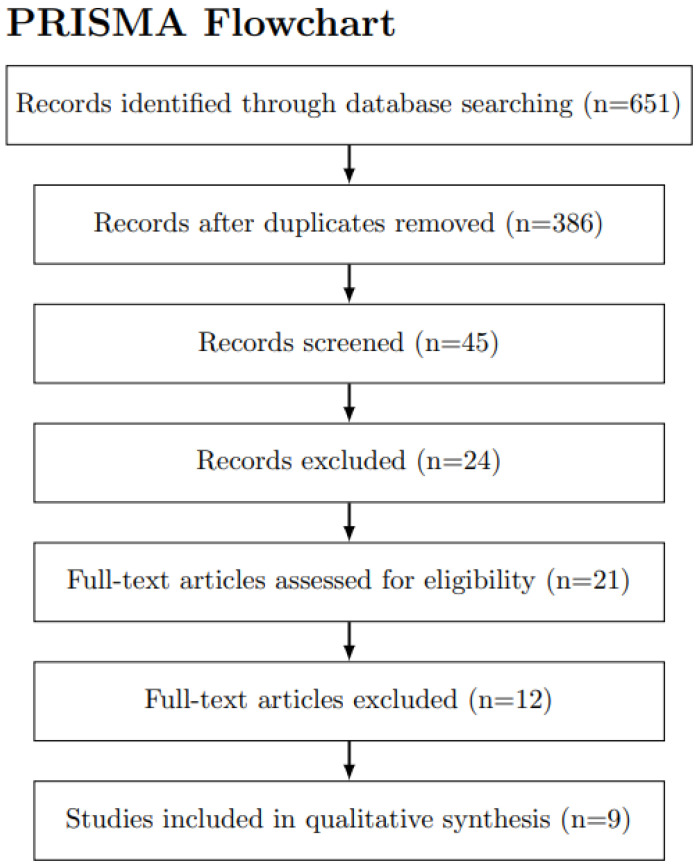
PRISMA Flowchart for the selection process of studies included in this review.

Original research articles, clinical trials, observational studies, and case series (*n* > 2) investigating CMV pharmacotherapy in critically ill patients with COVID-19 were included in our review. We excluded non-English articles, case reports, conference abstracts, editorials, and reviews. To determine eligibility, two independent reviewers screened the titles and abstracts of the identified articles. Disagreements were settled through discussion and consensus, with the assistance of a third reviewer if needed. The PRISMA flowchart (Figure 1) illustrates the step-by-step process of identifying, screening, and assessing the eligibility of studies for inclusion in this narrative review. Subsequently, a full-text review of the selected publications was conducted to extract relevant data on study design, sample size, patient population, intervention details (e.g., antiviral agent type and dose), comparison groups, and outcome measures. The findings from the included studies were qualitatively synthesized, with a focus on the effectiveness and safety of CMV pharmacotherapy in the management of COVID-19 critically ill patients. Our findings are comprehensively presented in Table 1 [6,8,11,12,13,14,15,16].

### 2.1. CMV Reactivation in Critical Illness

CMV reactivation is of significant concern in a variety of clinical scenarios, including bacterial sepsis, and following hematopoietic cell transplantation [17]. While CMV is notorious for causing considerable morbidity and death in immunocompromised individuals, research suggests that CMV reactivation is common in critically ill, immunocompetent patients in the ICU, and may be associated with worse outcomes [18], which has been verified by systematic reviews and meta-analyses in the literature. In a systematic review from 2008, Osawa et al. [19] found that CMV reactivation, defined as the detection of CMV in all types of samples, including blood, urine, and respiratory secretions, occurs in up to 36% (median 25%) of critically ill immunocompetent patients on mechanical ventilation or sepsis within 4–12 days following admission to the ICU; whether CMV reactivation was significantly associated with increased mortality or end-organ damage directly was not disclosed. In a more recent meta-analysis of 22 studies [20], CMV reactivation was associated with increased ICU mortality (odds ratio (OR), 2.55; 95% confidence interval (CI), 1.87–3.47), overall mortality (OR, 2.02; 95% CI, 1.60–2.56), as well as prolonged duration of mechanical ventilation and ICU length of stay. Indeed, observational studies have consistently shown a link between CMV reactivation and poor clinical outcomes for over a decade. In a prospective study, Limaye et al. [21] found that CMV reactivation was independently associated with prolonged hospitalization or death. In another prospective, double-blind study, Heininger et al. [22] proved that CMV reactivation was independently associated with increased length of stay in the ICU and hospital, prolonged mechanical ventilation, and impaired pulmonary gas exchange in patients with severe sepsis. In 2015, Frantzeskaki et al. [23] reported that CMV reactivation (defined as CMV DNAemia greater than or equal to 500 copies/mL) occurred in 13.75% of critically ill mechanically ventilated ICU, immunocompetent patients, and was associated with more severe organ dysfunction, rather than worse clinical outcomes. The most recent prospective study, including 71 patients in China, concluded that the incidence of CMV reactivation is around 18% in ICU patients, and it can lead to various adverse prognoses, including higher rates of complications, longer duration of mechanical ventilation, increased hospitalization costs, prolonged length of ICU stay, and increased 90-day all-cause mortality rate [24]. In the ICU population, steroid administration, prolonged mechanical ventilation, and sepsis have all been recognized as risk factors for CMV reactivation [19,25,26]. In a pooled analysis of prospective studies on the matter, Imlay et al. found that CMV reactivation at any level (>100 IU/mL, >1000 IU/mL as well as peak viral load) was independently associated with a variety of adverse clinical outcomes by day 28, including death and fewer ventilator-free days [27]. Evidently, CMV reactivation perplexes hospitalization and care in the ICU setting; however, it remains unclear whether CMV reactivation plays a causal role or if it is a surrogate for more severe illness. Imlay (2020) and Lachance (2017) [20,27] both suggest that randomized, placebo-controlled efficacy trials are needed to determine whether prevention of CMV reactivation improves clinical outcomes of patients with critical illness.

On the contrary, the association between CMV seropositivity and ICU outcomes is not well established. In a study involving over 1500 patients, Vlieger et al. [28] reports no association between cytomegalovirus serostatus and ICU mortality, in-hospital mortality, time to alive discharge from ICU and hospital, time to alive weaning from mechanical ventilation, or need for renal replacement therapy in non-immunocompromised patients with an ICU stay of 3 days or more. In a prospective study involving over 300 patients with ARDS, Ong et al. found that CMV seroprevalence was not associated with prolonged mechanical ventilation or increased mortality, except for patients presenting with septic shock [29].

### 2.2. CMV Reactivation in COVID-19 Patients

The role of CMV in the context of COVID-19 critical illness is the new area of interest. In a systematic review with meta-analysis of studies that evaluated active human herpesvirus (HHV) infection in COVID-19 patients, active CMV prevalence was between 19 and 25% [30,31]. CMV reactivation has the potential to exacerbate COVID-19 severity and lead to worse outcomes, such as increased mortality rates and prolonged hospitalization according to some case-series [32,33]. A study from Italy combining data from three ICUs [16] suggests that in-hospital mortality is indeed higher among patients with CMV reactivation compared to patients without CMV reactivation (67.0% vs. 24.5%). However, the adjusted Cox proportional hazards regression analysis, which considered relevant clinical covariates with unadjusted *p* value < 0.1, did not confirm this association (Adjusted HR: 1.141, 95% CI: 0.757–1.721, *p* = 0.528) [16]. Furthermore, a sensitivity analysis performed only in patients with CMV-related pneumonia treated with ganciclovir showed no independent association between CMV pneumonia and mortality at day 60 (HR: 1.248, 95% CI: 0.732–2.129, *p* = 0.415) [16]. Additionally, a secondary propensity score-matched analysis indicated no association between CMV blood reactivation and mortality at day 60 (HR: 1.105, 95% CI: 0.738–1.640, *p* = 0.638) [16].

Nonetheless, in the context of COVID-19, CMV reactivation may place a higher burden on healthcare systems due to the increased need for hospitalization and intensive care support. In immunocompetent critically ill patients with COVID-19, invasive mechanical ventilation and newly acquired bacterial infections have been identified as risk factors for CMV reactivation [16]. CMV-specific antibodies have emerged as strong predictors of COVID-19 severity [7]. CMV seropositivity is associated with increased odds of hospitalization (OR = 3.1, *p* < 0.001) and ICU admission risk (OR = 5.0, *p* < 0.001) independent of age and comorbidities. In addition, a pre-print study utilizing machine learning to predict the risk of COVID-19 infection and severity in 4510 adults found that CMV-specific antibodies were the strongest predictors of infection risk [34]. Moreover, CMV seropositivity has been shown to independently affect overall survival in allogeneic hematopoietic cell transplantation (allo-HCT) patients with COVID-19 [35]. The negative impact of CMV seropositivity on survival after hematopoietic cell transplantation is mediated by the patient’s serostatus, irrespective of the donor’s serostatus.

### 2.3. Understanding the Pathophysiology

Multiple biologically plausible mechanisms have been proposed to explain the link between CMV reactivation and worse clinical outcomes in critically ill patients with our without COVID-19. These mechanisms include primary lung injury, amplification of systemic and/or lung inflammation, and secondary immunosuppression. Papazian et al in a narrative review, suggests that CMV can be pathogenic in the ICU by direct organ insult, decreasing host defenses against other microorganisms, and exaggerating inflammatory response [36].

CMV reactivation has the potential to cause both direct or indirect lung injury. For instance, CMV is known to be a significant pathogen in lung transplantation cases, causing severe pneumonia through a direct cytopathic effect [37]. CMV has also been found to upregulate the SARS-CoV-2 receptor ACE2, which facilitates viral entry into cells [38]. This finding implies that local CMV reactivation in the airways may exacerbate lung injury in COVID-19 patients. ACE2 expression is also high in the intestinal tract, another prominent location for CMV reactivation in the critically ill, that has also been reported multiple times in the context of COVID-19.

Another mechanism by which CMV reactivation can negatively impact clinical outcomes is the amplification and/or further dysregulation of the systemic inflammation. Patients with COVID-19 who are CMV seropositive have been found to exhibit dysregulated systemic cytokine responses [39], including IL-6, IL-8, TNFα, and IL-10 concentrations, which are also elevated in patients with critical illness [40]. Intense and dysregulated inflammation, as well as compensatory anti-inflammatory responses, are speculated to facilitate secondary CMV reactivation in sepsis, but the specific mediators and pathogenesis remain undefined. Certain interleukins have been specifically associated with CMV reactivation in other populations. Cano et al. found that alleles of three cytokines, TGFβ1, IL-4, and IL-2, were significantly associated with CMV reactivation in cancer patients [41]. In lung transplant recipients, elevated levels of IL-10 in the bronchoalveolar lavage (BAL) and/or plasma were associated with delayed CMV clearance [42]. Furthermore, TNF-alpha and IL-1beta have been shown to be capable of reactivating CMV from latency in the lungs of previously healthy mice [43]. 

Multilevel immune activation and profound inflammation are thought to drive CMV reactivation in COVID-19 and lead to poorer clinical outcomes. Additionally, CMV can influence various immune system functions, including those of natural killer (NK) cells and T cells [44]. Recent studies have revealed that patients with severe COVID-19 exhibited higher levels of CMV-specific IgG and increased numbers of cells infected by SARS-CoV-2 in vitro [7]. Finally, both CMV and SARS-CoV-2 have been shown to induce the production of specific microRNAs (miRNAs) to aid in their replication cycles and regulate gene expression, which may vary depending on disease severity [45]. CMV miRNAs play essential roles in regulating CD34+ hematopoietic progenitor cells’ (HPCs) proliferation, hematopoiesis, and entry into and exit from latency [46]. CMV latency is established when peripheral monocytes seed infection to CD34+ hematopoietic stem cells (HSCs) in the bone marrow. Reactivation from latency involves the stimulation of pathways involved in cellular differentiation. Notably, SARS-CoV-2 has been observed to infect primary CD34+ HSCs, inducing ex vivo formation of defective erythroid and megakaryocytic cells that may become targets of humoral and adaptive immune cells [47], potentially mediating CMV reactivation.

A third mechanism linking CMV reactivation to clinical outcomes is secondary immunosuppression. CMV is known to encode a viral homolog of the im-mune-suppressive cytokine IL-10, which can modulate the host’s immune response and contribute to secondary immunosuppression in infected individuals [48]. Moreover, CMV can alter the expression of human leukocyte antigens, which play a crucial role in the immune system’s ability to recognize and eliminate infected cells [49]. This modulation could account for the fact that CMV reactivation is an identified risk factor for invasive pulmonary Aspergillosis and secondary bacterial infections in both COVID-19 and non-COVID-19 critical illness [16,50]. Altered memory T cell populations due to CMV infection can also impact the overall immune response against viral pathogens [51]. CMV reactivation in older individuals can result in immune dysregulation, particularly affecting T cell populations, which is characterized by an increase in CMV-specific memory and effector T cells and a concomitant decrease in the naive T cell pool. Consequently, older CMV-positive individuals may have difficulty generating an adaptive immune response to fight new infections, such as SARS-CoV-2. This immune dysregulation may contribute to the increased severity of COVID-19 observed in the elderly population, although not all SARS-CoV-2 variants exert the same effects on different age groups, as recent epidemiologic reports have illustrated the varying year-of-age specificity for COVID-19 variants in relation to mortality [52]. 

CMV’s role in exaggerating disease severity may, after all, be part of the bigger picture. For example, CMV seropositivity has been associated with cardiovascular comorbidities, like hypertension [53] and a higher incidence of thromboembolic events [54], both of which are linked to severe COVID-19 either in the form of predisposing risk factor or as a disease complication. According to a systematic review that assessed all published cases of patients with both SARS-CoV-2 and CMV coinfection, the most common end organ involved was the gastrointestinal tract, followed by the respiratory tract. Other findings of this study include a higher mortality risk in patients that required invasive mechanical ventilation and the fact that middle-aged or elderly patients with comorbidities are more likely to develop CMV coinfection [55]. Regarding the potential link between obesity, which is a significant risk factor for COVID-19, and CMV, the existing literature provides mixed evidence. Hamer et al. found no association between CMV seropositivity and obesity but did observe a weak association between CMV and metabolic dysfunction in non-obese adults [56]. Fleck-Derderian et al. reported no significant association between CMV and metabolic syndrome in adult females, regardless of BMI [57]. Froberg et al. demonstrated that CMV seropositivity was associated with higher serum total cholesterol levels in female patients under 50 years of age, but not in male patients of comparable age [58]. 

### 2.4. A Word in Diagnosis

Diagnostic evaluations are of paramount importance in order to carefully balance the potential benefits of CMV pharmacotherapy in COVID-19 patients, as there are risks associated with unnecessary treatment, such as adverse side effects and drug-drug interactions. Moreover, increased specificity of diagnostic procedures is crucial to identify subgroups at high risk for reactivation and subsequently select target populations for future interventional trials, as unnecessary treatment in patients without reactivation may cause harm without providing any benefit. As a matter of fact, the diagnosis of CMV is often overlooked in the ICU [59] due to the lack of awareness among doctors, and the inherent challenges in detecting the virus during its early reactivation stages, particularly in local sites such as the lung or bowel. Clinical diagnostics for CMV can be particularly challenging in these early stages, as traditional viremia tests may not adequately identify the virus. 

Polymerase chain reaction (PCR) assays in all types of samples are currently the standard diagnostic approach for detecting CMV in critically ill patients. Another diagnostic approach involves the CMV antigenemia assay, which detects the CMV protein pp65 on leukocytes using monoclonal antibodies, which is sensitive and able to quantify viral load, but has its shortcomings, given its methodology [18]. In a comparative study assessing the efficacy of both techniques, PCR detected CMV DNA in 54.6% of BAL samples and 12.0% of blood samples in patients with suspected CMV infection, while antigenemia was confirmed in 12.5% of blood samples [60,61]. In another study, Coisel et al. [62] found the diagnostic yield of BAL CMV PCR was 73% in comparison with the detection of CMV antigenemia, which was 46%, while Heininger et al. [22] reported slightly different diagnostic yields of tracheal aspirate CMV PCR (70%) versus blood CMV PCR (62%). In special populations, however, that the sensitivity and negative predictive value of CMV PCR may be suboptimal, as exhibited by Brantsaeter et al in an autopsy-based study [63].

It becomes evident that CMV reactivation rate depends on the studied population and the detection method. Research has shown that CMV reactivation rates have evolved over time, with reactivation occurring in 31% of CMV-seropositive patients when PCR was used [64]. In a 2009 meta-analysis, the rate of reactivation in CMV seropositive patients was 36% with PCR or antigen detection [65]. Papazian et al [36] reported that when depending on the methodology used, the incidence can reach 15–20% of ICU patients (20–40% in ICU patients with positive CMV serology). Early reports used viral cultures, which are less sensitive and slower than antigen and PCR testing [4]. 

To improve diagnostic accuracy, a variety of techniques have been employed. Bronchoalveolar lavage (BAL), for example, is a superior method for identifying CMV reactivation in the lungs through immunohistochemical staining of infected cells, although it may not always be feasible. Additionally, it is crucial to understand that PCR tests may not identify the virus in its early phase, particularly in blood samples. For instance, CMV colitis may be present in patients without CMV viremia, further complicating the diagnostic process. As such, it is essential to examine tissue samples by immunostaining or to perform PCR testing on respiratory or bowel samples to enhance the likelihood of detecting CMV. However, even in such case there is no consensus as to definitions of viremia with clinical significance or virus detection in the form of “innocent bystander” [4]. Thresholds for harmful viremia are lacking at the moment, hence strong indications for treatment initiation. Immunological assessments, such as IFN-γ produced by CMV-specific CD8+ T cells and NK cell function, might be a promising approach in the future to guide preemptive treatment in challenging cases [4]. 

Ultimately, the key to identifying CMV reactivation lies in actively searching for its presence, as the virus will not be found unless doctors make a concerted effort to look for it. By utilizing a comprehensive approach to CMV testing, healthcare professionals can more accurately diagnose the virus and make informed decisions regarding treatment for critically ill patients.

### 2.5. CMV Treatment and Prophylaxis in the ICU

Studies specifically addressing the efficacy of antiviral treatment in preventing or treating CMV reactivation in critically ill patients are lacking. However, treating patients with CMV- or HSV-specific disease and those with high viral load reactivation is generally considered appropriate. The potential benefit of early, preemptive treatment for patients with low viral load reactivation is still unclear and requires further investigation. Current CMV pharmacotherapies primarily include antiviral agents such as ganciclovir, valganciclovir, foscarnet, and cidofovir [65]. These medications target viral DNA replication by inhibiting the CMV DNA polymerase enzyme. Letermovir, a newer agent, inhibits the CMV terminase complex, preventing viral DNA cleavage and packaging. These drugs have demonstrated efficacy in the prevention and treatment of CMV infections in immunocompromised individuals, such as transplant recipients. 

While there is agreement on treating established CMV disease and providing prophylactic treatment for certain immunocompromised patients, such as solid organ [66] or allogenic bone marrow transplant recipients and patients with AIDS or low CD4 counts, no definitive criterion is available to guide which critically ill patients should be screened for CMV disease. An experimental animal study [67] assessing CMV prophylaxis reported that early initiation of antiviral therapy with ganciclovir at a dose of 10 mg/kg/day for 1–3 weeks is most effective in preventing sepsis-induced CMV reactivation and associated pulmonary injury in non-immunosuppressed hosts. Delaying therapy or decreasing the dose to 5 mg/kg/day allows significant breakthrough reactivation according to authors. This study also found that bacterial sepsis induces activation of lung-resident CMV-specific T-cells, which may contribute to sepsis-induced pulmonary injury in latently infected immunocompetent hosts.

Currently, there are only three randomized trials on CMV prevention in the ICU setting that have provided insights into the potential benefits and challenges of CMV pharmacotherapy. In the most recent one, Papazian et al. [68] failed to demonstrate a mortality benefit in mechanically ventilated (for more than 96 h) patients with CMV reactivation in blood with preemptive ganciclovir administration. On the other hand, a previous study from Cowley et al. demonstrated a reduction in CMV reactivation rates in mechanically ventilated patients treated with valacyclovir or valganciclovir [69], while the Limaye et al. [70] trial showed that preemptive ganciclovir administration increased ventilator-free days in critically ill patients. Notably, the Limaye et al. trial also employed a variety of statistical methods to assess the relationship between CMV reactivation and adverse clinical outcomes for which the authors reported a strong association, thereby suggesting a possible role for CMV prophylaxis in improving hospitalization outcomes. However, the Cowley et al. trial reported an increased mortality rate in the valacyclovir group compared to the control group, emphasizing the need to weigh potential risks and benefits when selecting and administering CMV pharmacotherapies in critically ill patients. Moreover, although both trials reported no significant differences in adverse events or safety outcomes between intervention and control groups, the safety and tolerability of CMV pharmacotherapies remain essential factors to consider in clinical practice, particularly in vulnerable, comorbid populations with complicated hospitalization courses.

To date, there is limited literature on the use of CMV pharmacotherapies specifically in COVID-19 patients. Studies have focused primarily on the relationship between CMV serostatus, reactivation, and COVID-19 outcomes rather than treatment per se, thereby conclusive evidence regarding the potential benefits or risks of CMV pharmacotherapy in critically ill COVID-19 patients remains lacking. In the only relevant study to date, Schoninger et all reports that there was no mortality benefit or any other clear clinical benefit in fact, to treating CMV reactivation in the COVID-19 ICU setting [11]. In another study from Italy, the ganciclovir-treated subgroup did not display an increased morality rate [16].

Antiviral treatment for CMV can carry risks, particularly in critically ill patients. Toxicity of ganciclovir, for example, includes granulocytopenia, anemia, thrombocytopenia, pancytopenia, and acute kidney injury. CMV-IVIG can cause adverse effects such as anaphylaxis/hypersensitivity reactions, aseptic meningitis, hemolysis, pulmonary edema, renal impairment, and thrombotic events [65]. As guidelines for treatment of CMV reactivation in non-transplant critically ill patients are not well established, it is important to weigh the risks and benefits carefully when considering CMV treatment in critically ill patients with COVID-19 pneumonia.

## 3. Conclusions

CMV pharmacotherapies may have a role in preventing or mitigating CMV reactivation in COVID-19 patients, particularly those who are at high risk for reactivation, such as CMV-seropositive individuals and immunocompromised patients. However, the current understanding of CMV reactivation in the context of COVID-19 is limited, and further research is needed to establish the efficacy and safety of CMV pharmacotherapy in this population. Future interventional trials could help determine whether CMV pharmacotherapies can improve clinical outcomes in COVID-19 patients at high risk for CMV reactivation. Finally, further investigation into the relationship between CMV reactivation and COVID-19 severity may provide valuable insights into the possible benefits of CMV-specific suppression and/or therapy in managing the disease and improving patient outcomes.

## Figures and Tables

**Table 1:** Summary of CMV Pharmacotherapy Studies in Critically Ill COVID-19 Patients.

Study/Citation	Type of Study	Country/Time Period	Patients Population and Severity Level (*n*)	IMV (%)	CMV Reactivation Rate (%)	Sample Type/Detection Cut-off/Recurrence of CMV Testing	Immunosuppression/Immunomodulatory Drugs Used	Viral Load/Organ System Manifestations	Mortality	Antiviral Treatment	Safety/Adverse Events of CMV Treatment
[Schoninger, Scott et al., doi:10.1093/ofid/ofac286]	Retrospective cohort study	United States/1 March 2020–30 April 2021	Critically ill adult COVID-19 patients with detected CMV viremia were admitted to MICUs (*n* = 80); treatment group (*n* = 43), control group (*n* = 37)	87.5% overall: treatment group (83.7%), control group (91.9%)	100%	Plasma, serum, or whole blood sample: Detection cut-off—CMV viral load ≥1000 copies/mL (positive) and CMV viral load <1000 copies/mL (low positivity)	Glucocorticoids: 99% of overall patients; treatment group (100%), control group (97%), *p*-value = 0.462 Median total dexamethasone dose equivalents: treatment group (309 mg), control group (188 mg), *p*-value = 0.017 Tocilizumab: 45% of overall patients; treatment group (51.2%), control group (37.8%), *p*-value = 0.333	Median maximum CMV viral load (IQR) was 1741 (308–8260) for the mortality group and 613 (183–1243) for the surviving group	Overall, in-hospital mortality—Treatment group: 37.2%, Control group: 43.2% (*p* = 0.749) ICU mortality—Treatment group: 37.2%, Control group: 37.8% (*p* = 0.954)	Treatment group received ganciclovir and/or valganciclovir for at least 5 days; Median duration of ganciclovir (IQR): 15 (8–27) days; Median duration of valganciclovir (IQR): 11 (7–15) days; Median duration of ganciclovir plus valganciclovir (IQR): 19 (9–30) days	Myelosuppression (defined as absolute neutrophil count (ANC) <1000 cells/μL (neutropenia) or <500 cells/μL (severe neutropenia) during the time period in which ganciclovir or valganciclovir was administered in a patient who previously had an ANC above these values before the start of ganciclovir or valganciclovir) was examined as a potential adverse event. No cases of myelosuppression in treatment group
[Yamamoto, Yuji, et al. doi:10.1002/jmv.27421]	Retrospective observational study	Japan/1 April–31 May 2021	59 patients with severe COVID-19 admitted to ICU	100%	25.4% (15 patients)	Plasma CMV-DNA/CMV-DNAemia > 200 IU/mL/Weekly follow-up measurements	Prior to admission, intensive immunosuppressive treatment was used in 8 patients (53.3%) with CMV infection and 14 patients (31.8%) without CMV infection. Of these, corticosteroid pulse therapy was administered to 5 patients (33.3%) with CMV infection and 8 patients (18.2%) without CMV infection. Tocilizumab was used in 3 patients (20.0%) with CMV infection and 6 patients (13.6%) without CMV infection. Baricitinib was administered to 3 patients (20.0%) with CMV infection and 3 patients (6.8%) without CMV infection.	Two patients with possible CMV gastrointestinal disease, two patients with possible CMV pneumonia	4 patients with CMV infection (26.7%), 0 patients without CMV infection	6 patients required antiviral treatment; 1 patient died due to possible CMV pneumonia	Not reported
[Talan L, et al. doi:10.4274/balkanmedj.galenos.2022.2022-2-2]	Retrospective Study	Turkey/March 2020–May 2021	218 ICU treated COVID-19 patients	100%	4.59% (10/218)	CMV viral load higher than 500 copies/mL	Corticosteroids (9/10 patients) Tocilizumab (4/10 patients)	Nonbacterial pneumonia	7/10	Standard CMV viremia treatment with ganciclovir or valganciclovir	Not reported
[Saade, A et al. doi:10.1016/j.idnow.2021.07.005]	Post-hoc analysis of a retrospective single-center study	France/30 February–10 May 2020	100 critically ill COVID-19 patients	54%	19% >3.5 log (significant reactivation) in 2 patients	Whole blood/Twice weekly and repeated in case of sepsis	All the information below concerned reactivation of all herpesviruses. Dexamethasone: 6 (16%) patients without viral reactivation and 27 (44%) patients with viral reactivation received dexamethasone. Eculizumab: 2 (5%) patients without viral reactivation and 8 (13%) patients with viral reactivation received eculizumab. Tocilizumab: 3 (8%) patients without viral reactivation and 2 (3%) patients with viral reactivation received tocilizumab.	One patient with esophagitis (CMV disease)	28% (ICU mortality) CMV reactivation was not associated with mortality (*p* = 0.31)	2 patients received valganciclovir (both solid organ transplant recipients). No specific outcomes reported	Not reported
[Simonnet, A et al. doi:10.1016/j.idnow.2021.01.005]	Single center retrospective study	France/16 March–6 August 2020	34 patients admitted to ICU for SARS-CoV-2-associated acute respiratory failure	88%	15% (5/34 patients) with CMV DNA detection	Quantitative PCR in whole blood, DNAemia detection was performed on average 3.7 times (range 1–15) per patient during ICU stay	Tocilizumab (3%), Corticosteroid treatment (88%)	Median blood viral load in patients with quantifiable CMV replication 4930 IU/mL (range 805–32,221)	18% (6/34 patients) died while in the ICU	3 patients with CMV reactivation were treated (ganciclovir in 2 cases, valganciclovir in 1 case) and were treated successfully	Not reported
[Naendrup, Jan-Hendrik et al. doi:10.1177/08850666211053990]	Retrospective single-center cohort study	Germany/March 2020–March 2021	117 patients with severe COVID-19 treated in ICU	Not reported	9% (11/117)	Whole blood, DNA levels higher than 1000 IU/mL	Systemic corticosteroid treatment (majority of CMV reactivations—55%)	Maximum viral copies in IU/mL for CMV reactivation: 4440 (1030–36,900)	ICU survival for CMV reactivation: 6/11 (55%)	Ganciclovir treatment in 6 patients (5/6, 83% survival); no treatment in 4 patients (0% survival); *p* = 0.048	Not reported
[Pérez-Granda, M J et al. doi:10.37201/req/068.2022]	Point prevalence study	Spain/30 February–10 May 2020	140 hospitalized adult patients with severe COVID-19; 26.42% (37 patients) ICU patients	19.28% (27 patients)	11.42% (16 patients); Patients with positive CMV viral load were mainly ICU patients (11/37–29.7%) while only 5/103 cases (4.85%) were hospitalized into general wards.	Plasma samples—threshold of detection: 31 IU/mL (20 copies/mL)	Tocilizumab (11.42%) for 16 patients Corticosteroids (prednisone) with a median dose of 225 mg	No specific manifestations mentioned. CMV viral loads ranged from 72 to 3126 IU, with a median of 328 IU (IQR 245.50 to 625.50)	13.57% (19 patients); In CMV positive cases death occurred in 8/16 cases (50%) compared with 11/124 (8.87%) in CMV negative patients (*p* < 0.001). CMV was an independent risk factor for death OR 12.31 (95% CI: 3.62–41.87, *p* < 0.001)	Ganciclovir treatment for 8 patients (5.71%), 6 of them CMV positive, none of them died	Not reported
[Niitsu, Takayuki et al. doi:10.1016/j.jinf.2021.07.004]	Retrospective study	Japan/April 2020-February 2021	Critically ill patients with COVID-19 requiring invasive mechanical ventilation for more than one week (*n* = 26)	100%	23.1% (6 patients)	CMV antigenemia assay for detecting pp65 antigen in peripheral blood leukocytes; CMV infection was defined as ≥1 antigen-positive cell per 50,000 leukocytes and two consecutive positive assays.	Corticosteroid use (100%)	CMV pneumonia in one patient (Case 1)	CMV group: 2 out of 6 patients (33.3%); Non-CMV group: 0 out of 20 patients (0%)	Ganciclovir therapy for CMV infection; two patients in the CMV group died from refractory respiratory failure, one of whom was diagnosed with CMV pneumonia.	Not reported
[Gatto, Ilenia et al. doi:10.1007/s00134-022-06716-y]	Prospective study	Italy/22 February–21 July 2021	431 patients with moderate to severe ARDS	64% (276 patients)	20.4% (88 patients)	Patients were screened at ICU admission and twice (in invasive mechanically ventilated patients). Quantitative CMV-DNAemia in the blood. Threshold > 62 UI/mL in whole blood	Steroids (methylprednisolone, dexamethasone)—91.4% (393 patients) Tocilizumab—82.6% (356 patients) Acyclovir prophylaxis—73.8% (318 patients) A larger proportion of patients with CMV reactivation received steroids (i.e., dexamethasone, methylprednisolone or both) (*p* = 0.005)	CMV-related pneumonia detected in 34.1% (30) of CMV reactivated patients. Median onset of 17 days (IQR 5–26) after ICU admission for CMV reactivation	The hospital mortality was larger (*p* < 0.001) in patients with CMV reactivation than without reactivation. No independent relationship between CMV pneumonia and mortality at day 60 (HR 1248; 95% CI: 0.732–2129; *p* = 0.415) was observed.	Ganciclovir treatment was given to 30 patients (6.9%) with CMV reactivation and clinical signs of CMV-related pneumonia. Among patients with CMV reactivation, patients with CMV-related pneumonia and treated with ganciclovir showed higher (*p* = 0.063) mortality (24/30; 80%) than patients without signs of CMV-related clinical pneumonia (35/58; 30%).	Not reported

## Data Availability

Not applicable.
